# Comparison between Different Structures of Suspended-Core Microstructured Optical Fibers for Volatiles Sensing

**DOI:** 10.3390/s18082523

**Published:** 2018-08-02

**Authors:** Diego Lopez-Torres, Aitor Lopez-Aldaba, Cesar Elosua, Jean L. Auguste, Rapahel Jamier, Philippe Roy, Manuel Lopez-Amo, Francisco J. Arregui

**Affiliations:** 1 Electric and Electronic Engineering Department, Universidad Publica de Navarra, Edif. Los Tejos, Campus Arrosadía, 31006 Pamplona, Spain; aitor.lopez@unavarra.es (A.L.-A.); cesar.elosua@unavarra.es (C.E.); mla@unavarra.es (M.L.-A.); parregui@unavarra.es (F.J.A.); 2 Institute of Smart Cities (ISC), Universidad Publica de Navarra. Campus Arrosadia, 31006 Pamplona, Spain; 3 XLIM Photonics Department, UMR 7252, University of Limoges, CNRS, F-87000 Limoges, France; jean-louis.auguste@xlim.fr (J.L.A.) raphael.jamier@xlim.fr (R.J.); philippe.roy@xlim.fr (P.R.)

**Keywords:** microstructured optical fiber (MOF), low-finesse Fabry–Pérot (FP), volatile organic compounds (VOCs), indium tin oxide (ITO), evanescent field

## Abstract

In this paper, different core structures of microstructured optical fibers (MOFs) for low-finesse Fabry–Pérot (FP) sensors are experimentally compared to get the highest sensitivity. These devices are designed for volatile organic compounds (VOCs) measurements. Indium tin oxide (ITO) thin films were deposited by sputtering on the MOFs and different optical fast Fourier transform (FFT) phase responses from the FP were measured for saturated atmospheres of ethanol. It has been demonstrated that the sensitivities of the developed sensors depend strongly on the geometry and the dimensions of the MOF-cores. The sensors show recovery times shorter than 100 s and the baselines are fully recovered after every exposure to ethanol vapors.

## 1. Introduction

Nowadays, the development of sensors based on fiber optics is experiencing considerable growth due to the relevance of the different applications where they are involved [1,2,3]. Another important factor in this growth is the advantages that optical fiber sensors offer over other kinds of sensors (for example, electronic sensors): small size, light weight, low cost, multiplexing or distributed measurements, remote monitoring, immunity to electromagnetic field interference, or no electrical biasing are some of them [4,5]. 

One specific type of fiber optic sensors is sensors based on microstructured optical fibers (MOFs) [6]. The geometry of MOFs is characterized by a periodic arrangement of air holes running along the entire length of the fiber, centered on a solid or hollow core [7]. The biggest attraction in these fibers is that by varying the size and location of the cladding holes or the core, the fiber transmission spectrum, mode shape, nonlinearity, dispersion, air filling fraction, and birefringence, among others, can be tuned to reach values that are not achievable with conventional optical fibers. Moreover, the existence of air holes gives the possibility of light propagation in air, or alternatively provides the ability to insert liquids or gases into the air holes. For these reasons, MOFs exhibit improved characteristics over conventional optical fibers and great potential for sensing applications [8]. 

As it has been mentioned in the previous paragraph, due to the geometry of MOFs, they can be divided into two different types: single material solid-core MOFs and hollow-core MOFs. More concretely, solid-core MOFs include one specific group of fiber named suspended-core MOFs. These fibers present relatively large air holes surrounding a small core (typically a few µms in diameter) which seem to be suspended along the fiber length but are maintained by small silica bridges. Due to the innate features of suspended-core MOFs, they have awakened the interest of many scientific groups and several papers have been published using them as curvature/bend sensors [9,10], displacement/strain sensors [11,12], pressure sensors [13,14], or temperature sensors [15,16], just to mention some. But in other cases, a sensing material is required to make the optical structure sensitive or aims to improve its MOF sensitivity [17]; this is the case of volatile organic compounds (VOCs) [18]. Suspended-core MOFs are a good option to detect VOCs because their specific geometry facilitates the absorption and desorption of VOC molecules and increases the evanescent field of the guided light: both effects enhance the total interaction of the optical signal with the analyte, improving the final sensitivity. 

Regarding sensing materials, metallic oxides, such as indium tin oxide (ITO), have been used in previous works to develop optical fiber optic sensors because they are able to detect organic vapors [19,20]. Metallic oxides and more concretely ITO, show reversible changes in their optical properties (refractive index, optical absorption) in the presence of VOCs [21,22]. In most cases, the operating mechanism of the sensors is based on intensity changes or wavelength shifts [23]. In this paper, the authors propose a reflection system for VOC measurements based on an ITO thin film deposited onto a microstructured optical fiber (MOF) low-finesse Fabry–Pérot (FP) using the fast Fourier transform (FFT) as the method of characterization of the sensor response [24,25]. FFT has been used in other works as an alternative method for the calibration and analysis of the sensor response: compared with spectral shifts of amplitude measurements, this one is more robust against artifacts and the resulting calibration is more linear [26]. 

Another important factor to be considered is that most of devices based on metallic oxides need to work at temperatures higher than 150 °C to obtain a proper sensitivity [27,28], but this paper proposes to perform the study at room temperature (25 °C). At this temperature, the interaction between ITO thin film and VOCs is due to the oxidation and reduction (chemisorption) reactions because of the presence of external agents (oxygen) that produce conductivity changes, as explained in References [29,30]. Both phenomena are transduced to a phase shift between the modes traveling through the core and the ones that are coupled to the evanescent field: in this manner spectral shifts in the interferometric pattern can be used to characterize the sensor response.

In brief, the synergy resulting from the combination of suspended-core MOF used to implement FP cavities together with metallic oxides is clear. One of the most important factors on the sensor sensitivity is the geometry of the MOF section as it determines the coupling to the evanescent field, and therefore, the interaction with the sensing material [31]. Considering this context, the authors propose (for the first time to our knowledge) to give an explanation, based on a new theoretical analysis and an experimental study, of why and how it is possible to improve the sensitivity and kinetics of previously published sensors by studying the effect of the different suspended-core MOFs geometries (MOFs with different cores and hole dimensions, and even introducing a hole in the center of the MOF core) on the final sensor sensitivity. As proof of concept and in order to verify this assumption, the different MOF structures were exposed to saturated atmospheres of a concrete VOC, ethanol.

## 2. Materials and Methods

### 2.1. Suspended-Core MOF Sensor Fabrication

**Figure 1:**
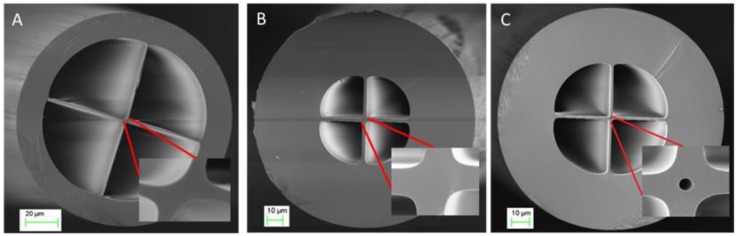
MOF-1 (**A**), MOF-2 (**B**), and MOF-3 (**C**) cross section. In every figure, the detail of the core geometry can be appreciated and especially in (C), the hole located in the center of the optical fiber. MOF = microstructured optical fibers.

Three different sensors were fabricated by splicing a single mode optical fiber (Corning SMF-28) to a segment, from 0.3 mm to 1 mm, of three new rectangular-shaped suspended-core MOFs to prepare low-finesse Fabry–Pérot interferometers: each one showing distinct geometries in terms of silica bridges and core. The suspended-core MOFs were fabricated using the stack and draw process [32]. Three different MOFs were employed: they are all formed by four large air holes divided by four bridges from the cladding to a rectangular core, but there are differences between them: MOF-1 showed a solid rectangle core of 2.62 μm by 1.02 μm (Figure 1A) whereas MOF-2 has a 3.1 μm by 4.4 μm core (Figure 1B); finally, MOF-3 also showed a rectangle core (2.73 μm by 4.2 μm) and a hole (with a diameter of 761.1 nm, Figure 1C) in its center. From now on, the fiber optic sensors will be named in the same way as the MOFs with which they have been made of, in order to identify them easily.

The low-finesse Fabry–Pérot cavities were made by splicing a single mode fiber to the MOF fibers. The MOFs were cleaved at the other end, creating the cavity with a length of ~500 μm. The splices were made using a commercial arc-electric fusion splicer machine, in manual mode. Since these MOF have a high air filling fraction, the typical arc power and duration used for single mode fibers fusion induces the MOF to collapse. Thus, a study was made on the arc power and duration settings in order to develop a new adapted program. This program allowed splicing the SMF and MOF without collapsing the MOF. To ensure that the SMF and MOF cores were aligned before splicing, the manual approximation of both fibers were made while illuminating the SMF and retrieving the signal at the MOF’s opposite end. Through the output at the MOF, it was possible to observe the amount of light transmitted from one core to the other.

A low-finesse Fabry–Pérot interferometer consists of two mirrors separated by a cavity with a length *d*. This cavity can be made by a MOF since its core refractive index is different from the SMF’s core refractive index. When splicing a piece of MOF to a SMF, two mirrors are formed at both ends of the MOF: (1) in the interface SMF–MOF due to the discontinuity in refractive index between both fibers and (2) at the interface MOF–air, since this high discontinuity provides Fresnel reflection (3.3%). The low-finesse Fabry–Pérot interferometer is created when a light beam enters the cavity (MOF) and is reflected between the interfaces (1) and (2). Each beam has a fixed phase difference with respect to the preceding one, and this phase difference corresponds to the extra path length travelled in the cavity. The interferometric signal of this cavity has a period corresponding to Δλ = λ2/(2*nd*), where λ is the wavelength of operation and *n* is the cavity refractive index.

### 2.2. Sensing Material

The sensing material selected was ITO. This metal oxide has a low selectivity towards VOCs [33]; this feature can be very useful if the sensor is included in a sensor array because it allows, with the use of post processing techniques such as principal component analysis (PCA) [34], the identification and the detection of VOCs’ complex mixtures [35]. Furthermore, due to the high sensitivity of ITO towards VOCs added to the study that this paper proposes, it is possible to detect VOCs in small concentrations that range from ppm down to ppb [36].

### 2.3. Experimental Set Up

ITO thin film was deposited onto the head and into the walls of the MOF suspended-core sensors using a sputtering machine (Pulsed DC sputtering System, Nadetech Innovations, Pamplona, Spain). The construction parameters were configured as follows: a partial pressure of argon of 5 × 10^−2^ mb, a current of 160 mA, and a voltage of 190 V for 8 min. The distance between the target of ITO and the head of the sensors was set at 6 cm. The suspended-core MOF sensors were placed into the sputtering machine as perpendicular as possible to the ITO target in order to obtain a homogeneous ITO thin film. 

**Figure 2:**
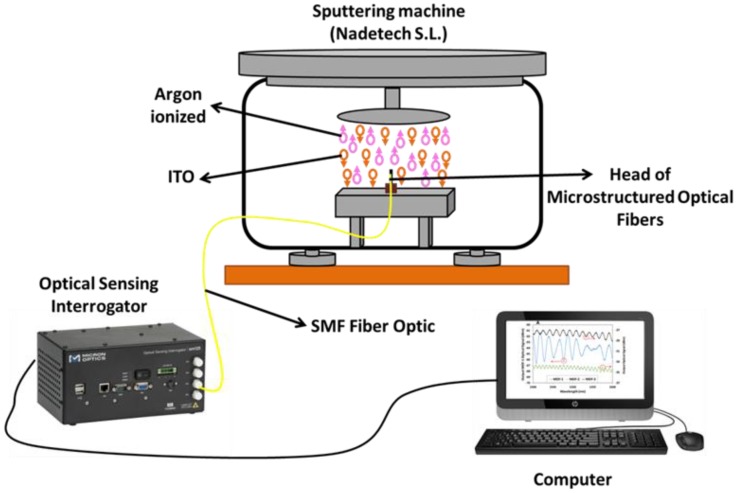
Experimental setup used to control the construction process of the different MOF sensors. ITO = indium tin oxide; SMF = single mode fiber.

Figure 2 shows the set up used during the construction process. It consists of a commercial optical interrogator for optical fiber sensors (Smartec SM 125, Lugano, Switzerland) and the sputtering machine that was described above. The optical interrogator was used to illuminate the MOF sensors and to record their reflected optical power; at the same time, their signals were processed with a computer using the commercial software Matlab^®^ (Mathworks, MA, USA). Due to this set up, it was possible to verify in real time that the amplitudes of the reflected optical power from the sensors were high enough to characterize the sensor response.

**Figure 3:**
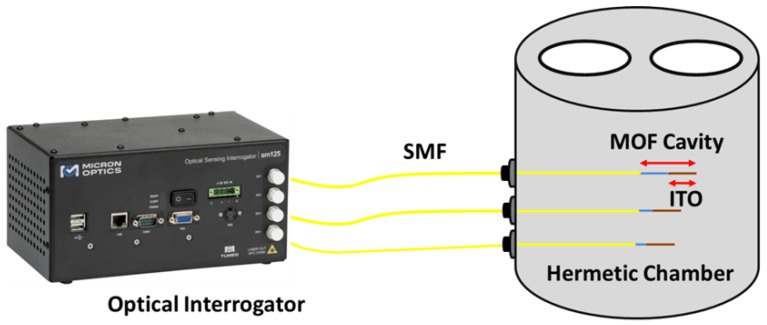
Experimental setup used to simultaneously analyze the response of the sensors towards saturated atmospheres of ethanol.

**Figure 4:**
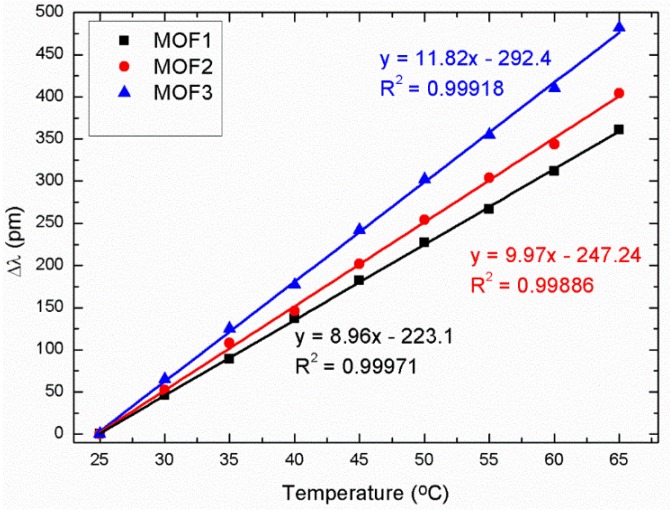
Characterization curves of the three different MOF sensors when they were exposed to temperatures from 25 °C to 65 °C.

The set up proposed to carry out the VOC measurements (see Figure 3) was an in-reflection one using the same commercial optical interrogator (Smartec SM 125). As explained at the beginning of the section, it was used both to illuminate the MOFs as well as to analyze the sensor responses; in this manner, it was possible to simultaneously measure the response of the three different sensors when they were exposed to VOCs with the same temperature and humidity values. These responses were performed placing the sensors in a hermetic metallic cell (avoiding any external contamination as, for example, external gases and ensuring constant humidity and temperature values during the measurement process). At this point, it is important to mention that all sensors used in this paper are sensitive to temperature as all of them were made of a MOF–FP cavity. Because of this reason, if the temperature varies during the exposure of the MOF sensors towards ethanol, it is necessary to use another sensor as a temperature reference in order to avoid the temperature crosstalk and its influence on the final sensor sensitivity, as Reference [37] proposes. Figure 4 shows the temperature characterization curves for the three sensors after the deposition process. Their sensitivities, when they were exposed towards temperature from 25 °C to 65 °C, were: MOF-1: 9.025 pm/°C; MOF-2: 10.1 pm/°C; MOF-3: 12.05 pm/°C.

The volume of the hermetic chamber was calculated in order to inject a controlled volume of ethanol that saturated the atmosphere of the chamber. Ethanol was injected with a pipette through the hermetic chamber holes. After 10 min of exposure, the ethanol injected was evaporated and then, the door of the hermetic chamber was opened for a period of 400 s, with the aim of ensuring that ethanol was removed of the hermetic chamber by applying a nitrogen flow. 

### 2.4. Parameters and Software Used in the Theoretical Simulations 

The finite element method (FEM) is the method selected to make the theoretical simulations of the different MOF structures. Each single structure was designed and computed with COMSOL© Multiphysics software and evaluated with COMSOL© Wave Optics Module. For every simulation, silica refractive index was set at 1.444 and air (holes inside the fiber) at 1. In this work, many high order modes have been calculated for all the studied structures, although only the fundamental mode, LP_01_ at 1550 nm is shown for the sake of simplicity of interpretation. This simplification is due to the power difference between the fundamental mode and the high order ones (one order of magnitude) that makes their influence negligible. All fibers are excited by the same Gaussian beam (corresponding to the LP_01_ of a SMF). These simulations were performed in a two-step analysis: firstly, the fundamental mode of the MOF was obtained and used as input in the second step. The second step was a propagation study where the maximum electric field values were obtained in order to compare the performance of the different structures presented. Analogously to an SMF optical fiber, LP_01_ is the lowest order propagated mode with the highest effective refractive index and which presents [38], in a symmetric SMF, a Gaussian optical power profile with a maximum in its center. 

To study and verify the fibers’ performance as optical waveguides and the effect of the ITO thin film in their behaviors, new field simulations were performed taking this new material into account. The complex refractive index of ITO thin film was measured using an ellipsometer (UVISEL 2, Horiba) and the result was 1.81103 + j0.051964 at 1550 nm (this exact value was used during the ITO simulations).

For these new simulations, symmetric and homogeneous fiber structures (in practice, they show slight variations due to the fabrication process) as well as thin films deposited by means of sputtering technique were implemented in order to achieve symmetric distributions of the LP_01_ fundamental mode.

## 3. Results and Discussions

### 3.1. Deposition ITO Procedure

**Figure 5:**
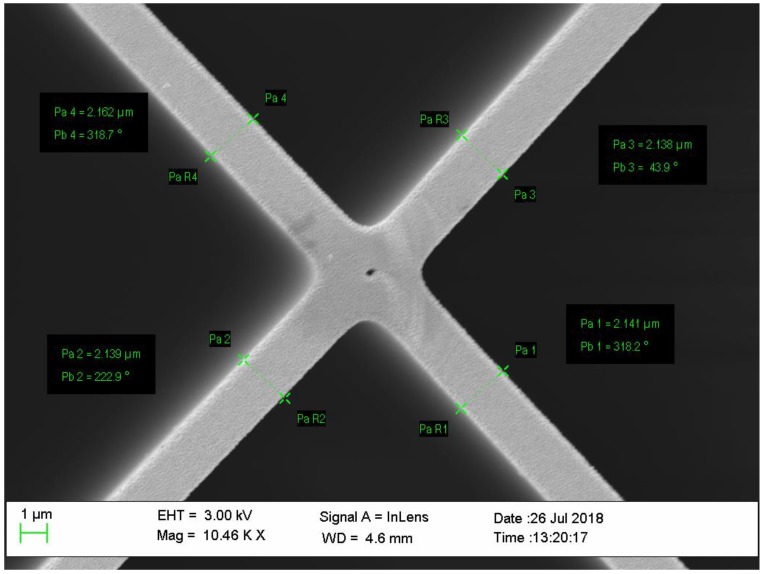
ITO nanofilm thickness measured for the MOF-3 using the SEM after 8 min of sputtering construction process.

To prepare the different MOF sensors, ITO nanofilms were deposited following the procedure explained in the Section 2.3. At this point, it is very important to remark that the goal of this paper was to improve the sensitivity of the MOF sensors only by means of its structures; the effect of other important factors on the MOF sensor sensitivity, such as the thickness of the nanofilm, will be studied in future papers. For this reason, and in order to obtain the same thickness for every sensor, sputtering technique was selected. This technique allows the final thin film thickness to be controlled with high accuracy and it can be considered a reproducible process [39], but the structure of the MOFs (core and bridges) do not always present the same dimensions (due to the custom fabrication process). The construction parameters were set based on previous studies with the intention of depositing a thickness which provides an optimal sensitivity. These parameters were the same for the three fibers (see Section 2.3), so that for a certain sputtering time, the final thickness was assumed to be similar in the three cases. Following these considerations, the film deposited onto MOF-3 was determined from images captured by a scanning electron microscope as follows: firstly, the width of each of the four bridges of MOF-3 was measured and averaged (1.158 ± 0.009 µm) prior to the coating deposition in order to minimize the possible error in this measurement; secondly, after the sputtering deposition, the average width of the four bridges was measured again (see Figure 5). To determine the real value of the MOF-3 ITO nanofilm thickness, both values were subtracted (average width before and after deposition); the result obtained was 500 ± 17 nm. 

### 3.2. Suspended-Core MOF Field Distribution Simulations

Theoretically, when the core of a suspended-core MOF is smaller, the interaction of the evanescent field with the surrounding external medium is stronger because the modes travel along the fiber less confined; on the other hand, although this structure magnifies this effect, the propagated optical power is lower when the core effective area is reduced. Therefore, there is a tradeoff between evanescent field interaction and coupled optical power. 

With the purpose to study the effect of the suspended-core fiber geometry on the coupled optical power and on the evanescent guided field of light, mode field distributions were simulated for the three different types of fiber (without and with ITO nanofilm) as described in Section 2.4.

#### 3.2.1. Theoretical Simulations without ITO Nanofilm Deposited

**Figure 6:**
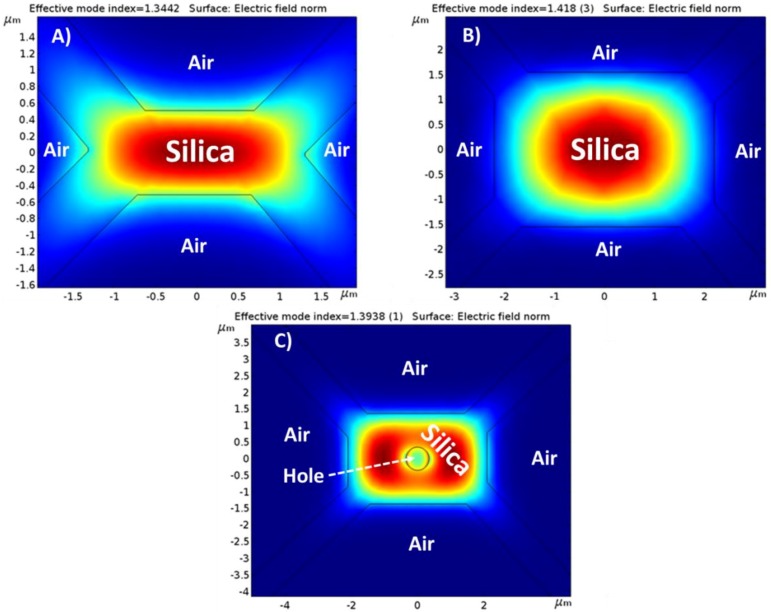
LP_01_ modal field distribution for fiber MOF-1 (**A**), MOF-2 (**B**), and MOF-3 (**C**).

In this section, the results obtained for simulations without ITO nanofilm deposited are shown according to Section 2.4. Figure 6A shows the LP_01_ mode propagated through fiber MOF-1. Due to the reduced dimensions of MOF-1’s core, on the one hand, low optical power is guided in the core showing maximum values of 50 V/m. On the other hand, this optical power has low confinement showing an evanescent field outside the core reaching values of 38 V/m. LP_01_ mode presents an effective refractive index of 1.344. As expected, the effective refractive index of the LP_01_ is affected by the guiding structure, and more precisely by the effective area of the core cross section; if this area is reduced, the value of the effective refractive index is decreased. Likewise, the fundamental mode field distribution was simulated for MOF-2 as Figure 6B illustrates. As it can be noticed, this particular fiber with its rectangular core presents bigger dimensions in comparison with MOF-1 and therefore, the coupled optical power is higher (reaching maximums of 340 V/m) and more confined in the core. Although, theoretically, the evanescent field should be weaker in MOF-2 than in MOF-1, due to this important difference in the guided optical power, a stronger evanescent field (100 V/m) is observed outside the core. The LP_01_ effective refractive index is higher in MOF-2 (1.418) than in MOF-1 due to the increase of the silica-core surface.

Finally, LP_01_ mode field distributions for MOF-3 are displayed in Figure 6C. This fiber presents almost similar dimensions as MOF-2 but it includes a small hole of 761 nm diameter located at the center of the silica core. The effect of this hole is clear: firstly, it produces a reduction of the optical power coupled to the MOF-3 core showing maximum values of 50 V/m (like MOF-1 values but 7 times less than MOF-2); secondly, it also produces an LP_01_ mode that travels along MOF-3 with less confinement producing an increase on its evanescent field values, reaching maximums of 33 V/m in the zone close to the hole. Moreover, an effective index decrease (1.394) can be noticed in relation to MOF-2 and this is due to the air hole inside the core which produces a reduction of the overall effective refractive index. 

In brief, based on these simulations, the interaction between the evanescent field of the guided/coupled light along the MOFs and a material deposited into the MOF walls, as well as on the top of it, could be possible if a film in the range of a nanometer [38] is deposited. But this interaction does not occur in the same way for all the studied structures. MOF-2 presents the highest values in terms of coupled optical power and evanescent field but the part of the evanescent field that interacts with the nanofilm is very small and it can be a drawback. MOF-1 shows the lowest values of coupled optic power and evanescent field and for this reason, this structure does not look like the best option to develop the sensor. Finally, MOF-3 core hole produces a different distribution of the guided optical power making its use very interesting for the final purpose of the sensor. It is true that the MOF-3 evanescent field values are lower than MOF-2, but the part of the evanescent field that could interact with the nanofilm is increased due to the effect of the hole; this phenomenon could make the sensitivity of the sensor improve. 

#### 3.2.2. Theoretical Simulations with an ITO Nanofilm 

The results of the theoretical simulations when an ideal homogeneous ITO thin film of 500 nm is deposited are presented in this section following the steps explained in Section 2.4. 

**Figure 7:**
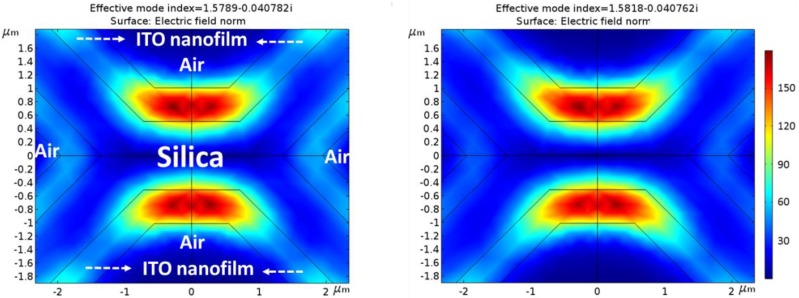
Mode field distribution for MOF-1 with ITO thin film. LP_01_ and LP_11_ are present simultaneously in each polarization.

Figure 7 illustrates the new optical field mode distribution of MOF-1 with ITO nanofilm. As it can be directly inferred from Figure 7, the waveguide properties have been highly modified: the light is no more guided by the silica structure but the ITO thin film, becoming a one layer antirresonant optical waveguide (ARROW) [40]. Since ncore < ncladding (where core is made of silica and cladding of ITO), total internal reflection does not occur at the core–cladding interface, and therefore no guided modes can propagate in the low-index core. However, these structures would support leaky-mode propagation [41]. In addition to this, Figure 7 indicates that not only the fundamental LP_01_ (symmetric) mode is being propagated through the fiber in the shown cases but also the LP_11_ (asymmetric) mode; LP_11_ propagated mode presents an asymmetric distribution with orthogonal phases between maximums in the same location as LP_01_. Generally, LP_11_ presents lower optical power (one order of magnitude) than LP_01_, but in this case, due to the dimensions of the new propagating structure, both optical powers are closer to interfering significantly with each other. As expected in ARROW structures, LP_01_ and LP_11_ can present very similar effective refractive indices [42] and appear simultaneously interfering one with the other. This is due to the dimensions of the fiber and the presence of two guiding areas isolated through a non-guiding region (in this case the original silica core). Both regions propagate the LP_01_ and LP_11_ modes within the same effective refractive index and therefore, they appear simultaneously interfering with each other. As it can be noticed, the effective refractive index of the propagated modes has been significantly increased as expected due to the higher refractive index of the ITO waveguide thin film. Moreover, the optical power of the guided modes has also been increased up to values of 147 V/m with evanescent fields of 70 V/m regarding the MOF-1 values obtained without ITO thin film (see Figure 6A). The former evanescent field (MOF-1 without ITO deposition) couples inside the ITO nanofilm and is propagated along the deposition, forming the new ARROW waveguide. 

**Figure 8:**
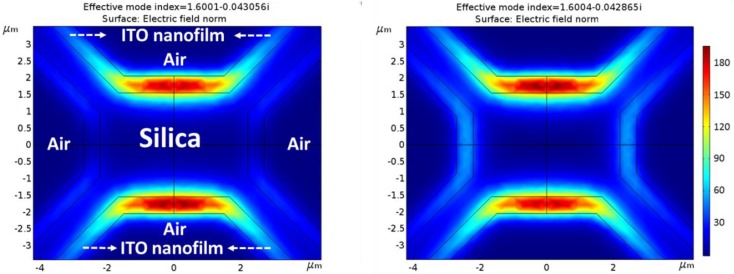
Mode field distribution for MOF-2 with ITO thin film. LP_01_ and LP_11_ are present simultaneously in each polarization.

MOF-2 also presents higher propagated optical power than MOF-1 (maximum of 181 V/m) when ITO thin film is deposited, as shown in Figure 8. This is due to the stronger field of the original MOF-2 structure (without ITO thin film deposited) despite its bigger core dimensions (Figure 6B). MOF-2 presents evanescent fields close to 90 V/m. It must be remarked, that in the particular case of MOF-2, the propagated optical power through the ITO thin film is lower than the original one (without ITO thin film). This is a result of the significant guiding effective area decrease: 3.1 μm by 4.4 μm for MOF-2 without ITO (dimensions of the core) and 0.5 μm (the thickness of the nanofilm) by 3 μm (the length of the bridges) for MOF-2 with ITO as now, the ITO thin film is the waveguide.

**Figure 9:**
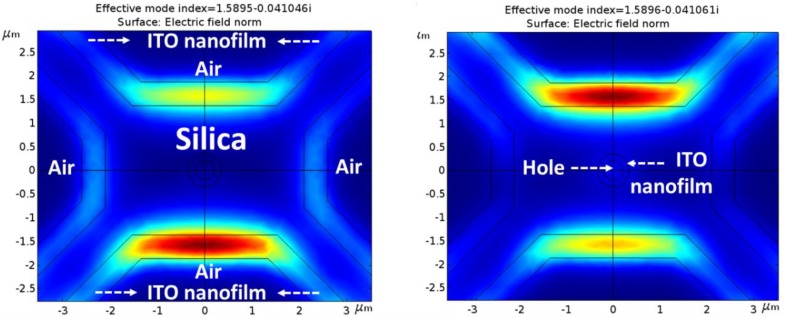
Mode field distribution for MOF-3 with ITO thin film. LP_01_ and LP_11_ are present simultaneously in each polarization.

Finally, when ITO thin film is deposited, MOF-3 presents the highest propagated optical power values (up to 340 V/m) and also the most powerful evanescent field reaching amplitudes of 200 V/m, as Figure 9 illustrates. In this case, the low confinement of the optical power (due to the air hole at the center of the silica core) in the original MOF-3 (without ITO thin film) allows higher optical values to be coupled into the ITO thin film waveguide. 

In order to simplify the data obtained in every simulation, Table 1 shows the maximum values of coupled optical power and evanescent field obtained for the different MOF structures with and without ITO thin film deposited. It is important to clarify that the optical power coupled to the ITO thin films does not only depend on the evanescent field of the MOF structures without ITO, but also on the part of optic power that this structure can be coupled to the ITO thin film which is inversely related to the MOF structure confinement.

Based on this, when ITO thin film is deposited, MOF-3 is the structure with the best conditions to develop the final ethanol sensor due to the hole effects; this hole causes the light to travel in a less confined manner making it possible to couple more optical power into the ITO thin film in comparison with MOF-2 (see the values of MOF-2 and MOF-3, before and after ITO deposition). According with this fact, the value of MOF-3 evanescent field is also increased and in this manner, the sensitivity towards ethanol should also increase. 

### 3.3. Ethanol Measurements 

**Figure 10:**
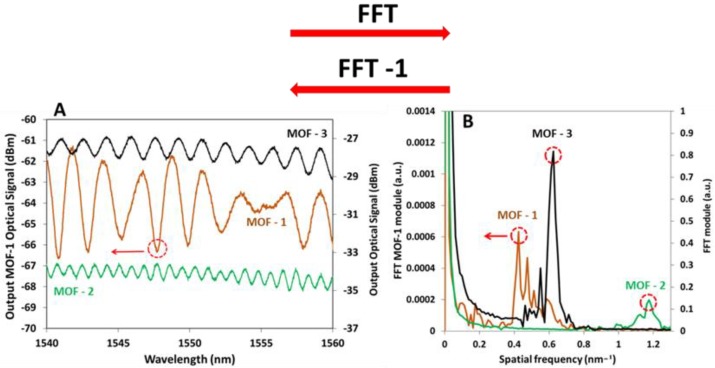
(**A**) Optical spectrum of MOFs and their fast Fourier transform (FFTs) after ITO deposition; (**B**) in both figures, the vertical left axis is applied for MOF-1 and the right axis for MOF-2 and MOF-3.

The sensors were characterized toward saturated atmosphere of ethanol using the fast Fourier transform (FFT) [39]; it is possible to apply this technique because the output optical signals of the three MOFs obtained after ITO deposition process describe a periodic sinusoidal function (see Figure 10A). The distance between peaks in the interference pattern is very small (a few nanometers) and this fact makes it very difficult to use traditional techniques based on registering the wavelength shift of remarkable points of the interferometric spectral response, such as a transmission valley. As can be observed in Figure 10B, the FFTs of the three MOFs’ optical spectrum present a principal component in the transformed domain, which corresponds to the main FP interference frequency.

Saturated atmosphere of ethanol changes the refractive index of the sensing layer (due to the reduction and oxidation (chemisorption) reactions that occur between ITO, oxygen, and ethanol); when it happens, the optical path of the transmitted light changes and consequently, a phase shift occurs. Based on this fact, the sensor responses were analyzed monitoring the phase shifts of these components using the set up explained in Figure 4. In Figure 10B, these components are encircled with a red dotted line. Additional interference frequencies can also be noticed for each MOF, corresponding to interferences involving higher order modes that transport lower optical powers. Also using the FFT, the wavelength shifts in the optical spectrum can be monitored without the effects of the noise influence or signal amplitude variations that other techniques as wavelength shifts have. 

Based on Reference [17], where the penetration depth of sputtering deposition (the length of the MOF with ITO) was 228 µm, and due to the smaller dimensions of the MOF holes used in this work, the authors assume that the penetration depth of the sputtering deposition is constant and FP cavities (MOF-1 350 µm, MOF-2 528 µm, and MOF-3 1 mm) are not completely covered in any of the cases by ITO; in this way, the different length of the FP cavities is a parameter which does not influence the sensor’s sensitivity towards ethanol.

**Figure 11:**
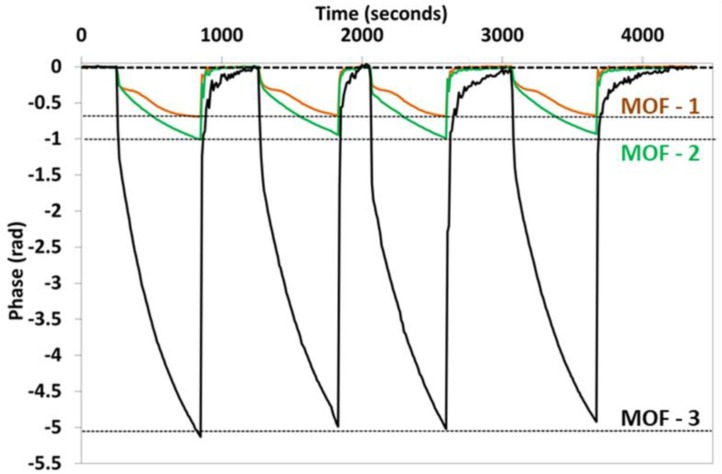
Phase responses of the three sensors for saturated atmospheres of ethanol.

With the aim of confirming the ideas that are derived from previous theoretical studies, every sensor was exposed to saturated atmospheres of ethanol; the three MOF sensors showed different FFT phase spectral shift responses (see Figure 11). In the first case, MOF-1 showed a maximum variation of 0.685 radians, which implies the lowest sensitivity among the sensors. As previously mentioned, it is a consequence of the dimensions of the core; it has an original low evanescent field power which makes it difficult to couple light into the ITO thin film. Sensors fabricated with MOF-2 and MOF-3, with wider cores, showed higher sensitivities. Although the external dimensions of the cores of these last fibers are similar, the maxima phase shift obtained for MOF-3 (5 radians) is five times greater than the maxima phase shift obtained for MOF-2 (1 radian) and four times greater than previous papers published when MOFs are used to detect VOCs [43]. This result is in accordance with the results of the theoretical study performed in previous sections and reinforces the hypothesis that the increment in the sensor sensitivity is produced by the effect of the MOF-3 core hole. This hole produces a loss of light confinement and an increase in the evanescent field power (in comparison with the case of MOF-2). Due to this fact, when this power (the evanescent field power of MOF-3 without ITO thin film) is coupled to ITO thin film, the penetration depth of this evanescent field is increased and consequently, its interaction with ethanol occurs in a more optimal way. Furthermore, the sensors’ responses were repetitive, and every sensor always reached the baseline in a short period of time (below 100 s) after every exposure to ethanol.

## 4. Conclusions

In this paper, different optical sensing heads based on microstructured optical fibers (MOFs) have been designed and analyzed both theoretically and experimentally. The sensor heads were developed for VOC detection and tested in ethanol saturated atmospheres. It has been proved that there exists a tradeoff between the optical power guided by the original MOF (without ITO thin film depositions) and the mode distribution confinement. This tradeoff determines the optical power of the propagated modes in the MOF–ITO ARROW structure: geometries with small core dimensions lead to weaker core modes (less coupled optical power) but they show high evanescent field power because the light travels with less confinement; on the other hand, wider core structures lead to more powerful guided modes but having more confined distributions and lower evanescent field power.

It is important to comment that all the results inferred in this paper are not only based on the field amplitude results. These results are a strong support to the authors’ proposal: the sensitivity of these kind of sensors can be improved by means of the substrate fiber structure. Additionally, there is another important factor that was not mentioned: the diffusion of the VOCs in the thin film layer. In the case of this paper, as the ITO thickness deposited on every sensor is similar, the influence of this parameter on the sensor sensitivity has not been studied. Still, the authors think that the nanofilm thickness of the sensing layer plays an important role on the sensor sensitivity and for this reason, in future works, the influence of this parameter will be analyzed in order to deposit a suitable thickness, improving in this manner the sensitivity obtained in this paper.

When an ITO thin film is deposited onto the MOFs, the guidance of the light is altered: the signal travels through the thin film deposited instead of the silica core of the fiber. This new optical power and its new evanescent field determine every sensor’s sensitivity towards refractive index variations in the surrounding environment. Moreover, it has been demonstrated that the inclusion of a hole at the center of the fiber core (the structure of the MOF-3) makes the light travel in a less confined manner increasing the values of the new ITO-silica waveguide in terms of coupled optical power and evanescent field. Therefore, the sensitivity of this fiber, MOF-3, is also increased and in the case studied in this paper, the achieved sensitivity is enhanced by a factor of 5.

## Figures and Tables

**Table 1:** Maximum values of confined optic power and evanescent field obtained for the different microstructured optical fiber (MOF) sensors with and without indium tin oxide (ITO) thin film deposited.

		Maximum Optical Field (V/M)	Maximum Evanescent Field (V/M)
**MOF-1**	WITHOUT ITO	50	38
	WITH ITO	147	70
**MOF-2**	WITHOUT ITO	340	100
	WITH ITO	181	90
**MOF-3**	WITHOUT ITO	63	40
	WITH ITO	340	200
